# Rationale and design of a cardiac safety study for reduced cardiotoxicity surveillance during HER2-targeted therapy

**DOI:** 10.1186/s40959-023-00163-4

**Published:** 2023-03-09

**Authors:** Anthony F. Yu, Chau T. Dang, Justine Jorgensen, Chaya S. Moskowitz, Patricia DeFusco, Eric Oligino, Kevin C. Oeffinger, Jennifer E. Liu, Richard M. Steingart

**Affiliations:** 1 Department of Medicine, Cardiology Service, Memorial Sloan Kettering Cancer Center, 1275 York Avenue, New York, NY 10065 USA; 2 Weill Cornell Medical College, New York, NY USA; 3 Department of Epidemiology and Biostatistics, Memorial Sloan Kettering Cancer Center, New York, USA; 4 Hartford Healthcare, Hartford, CT USA; 5 Duke Cancer Institute, Durham, NC USA

**Keywords:** Cardiotoxicity, Cardio-oncology, Breast cancer, Echocardiography

## Abstract

**Background:**

Echocardiograms are recommended every 3 months in patients receiving human epidermal growth factor 2 (HER2)-targeted therapy for surveillance of left ventricular ejection fraction (LVEF). Efforts to tailor treatment for HER2-positive breast cancer have led to greater use of non-anthracycline regimens that are associated with lower cardiotoxicity risk, raising into question the need for frequent cardiotoxicity surveillance for these patients. This study seeks to evaluate whether less frequent cardiotoxicity surveillance (every 6 months) is safe for patients receiving a non-anthracycline HER2-targeted treatment regimen.

**Methods/design:**

We will enroll 190 women with histologically confirmed HER2-positive breast cancer scheduled to receive a non-anthracycline HER2-targeted treatment regimen for a minimum of 12 months. All participants will undergo echocardiograms before and 6-, 12-, and 18-months after initiation of HER2-targeted treatment. The primary composite outcome is symptomatic heart failure (New York Heart Association class III or IV) or death from cardiovascular causes. Secondary outcomes include: 1) echocardiographic indices of left ventricular systolic function; 2) incidence of cardiotoxicity, defined by a ≥ 10% absolute reduction in left ventricular ejection fraction (LVEF) from baseline to < 53%; and 3) incidence of early interruption of HER2-targeted therapy.

**Conclusions:**

To our knowledge, this will be the first prospective study of a risk-based approach to cardiotoxicity surveillance. We expect findings from this study will inform the development of updated clinical practice guidelines to improve cardiotoxicity surveillance practices during HER2-positive breast cancer treatment.

**Trial registration:**

The trial was registered in the ClinicalTrials.gov registry (identifier NCT03983382) on June 12, 2019.

## Background

Treatment with trastuzumab (Herceptin) or other human epidermal growth factor receptor 2 (HER2)-targeted treatment regimens reduces the risk of cancer recurrence and all-cause death in patients with HER2-positive breast cancer but is associated with cardiotoxicity, which manifests as clinical heart failure or left ventricular ejection fraction (LVEF) decline [1–3]. In clinical trials of sequential treatment with anthracycline chemotherapy followed by trastuzumab, 0.5–4.1% developed clinical heart failure and up to 18.6% developed a significant decline in left ventricular ejection fraction (LVEF) [1–4]. Thus, in the Food and Drug Administration (FDA) package insert for trastuzumab, routine LVEF assessments are recommended at baseline and every 3 months for patients receiving trastuzumab [5]. This recommendation has subsequently been supported by consensus documents from several professional societies [6–8].

Anthracycline chemotherapy has been a mainstay of breast cancer treatment since the 1980s, based upon early clinical trials showing that anthracycline-based treatment reduces breast cancer recurrence and improves survival compared to non-anthracycline regimens [9]. However, treatment with anthracyclines is an independent risk factor for cardiotoxicity during HER2-targeted therapy [10–12]. Data from several recent studies including the APT [13], BCIRG 006 [3], TRAIN-2 [14], and CLEOPATRA [15] trials provide evidence to support non-anthracycline based regimens as alternative treatments for many patients with stage I-IV HER2-positive disease. Importantly, non-anthracycline HER2-targeted treatment regimens have been associated with lower risk of cardiotoxicity [13, 16, 17]. These data have led to a trend of de-escalating therapy for some patients with HER2-positive breast cancer and an increase in the use of non-anthracycline regimens [3, 18].

Despite the changing landscape of HER2-targeted treatment, recommendations for cardiotoxicity surveillance during HER2-positive breast cancer treatment have not been updated. A one-size-fits-all approach of LVEF assessments every 3 months remains the standard-of-care for patients treated with HER2-targeted therapy, as recommended by the original FDA label for trastuzumab [5]. There is a need to revise published recommendations for cardiotoxicity surveillance, such that more frequent cardiac imaging is reserved for patients at high risk for cardiotoxicity and less frequent imaging is allowed for patients at low risk, [19] however data on the cardiac safety of this approach is lacking.

To address this knowledge gap, we designed a study to evaluate the cardiac safety of reduced cardiotoxicity surveillance in patients with HER2-positive breast cancer treated with a non-anthracycline chemotherapy regimen. We hypothesize that LVEF assessments can be safely reduced to 6-month intervals for patients at low risk for cardiotoxicity treated with a non-anthracycline regimen. We anticipate that findings from this study will provide the evidence needed to inform the development of a tailored and risk-based approach to cardiotoxicity surveillance.

## Methods

### Design and objectives

This is a single-arm prospective trial (ClinicalTrials.gov Identifier: NCT03983382) with a primary objective to evaluate the cardiac safety of a reduced cardiotoxicity surveillance strategy (every 6 months) in patients with HER2-positive breast cancer treated with a non-anthracycline HER2-targeted treatment regimen. Secondary objectives include the following: 1) to measure the change in LVEF and global longitudinal strain (GLS) after 6 and 12 months of treatment compared to baseline; 2) to estimate the incidence of asymptomatic LVEF decline; 3) to estimate the incidence of early interruption of HER2-targeted treatment; and 4) to determine feasibility of a reduced cardiotoxicity surveillance strategy.

### Participants and setting

The study will be conducted in HER2-positive breast cancer patients (stage I-IV) receiving a non-anthracycline-based chemotherapy regimen in combination with a HER2-targeted agent (e.g. trastuzumab, pertuzumab, or ado-trastuzumab emtansine). The primary exclusion criteria are: (1) prior treatment with anthracyclines or HER2-targeted therapy; (2) baseline LVEF < 53% (or institutional lower limit of normal); (3) systolic or diastolic blood pressure ≥ 160 mmHg or ≥ 90 mmHg, respectively; and (4) history of heart failure, cardiomyopathy, or other significant CVD associated with increased cardiotoxicity risk (e.g. atrial fibrillation, atherosclerotic cardiovascular disease, significant valvular heart disease, etc.). Following approval by the primary medical oncologist, written informed consent will be obtained from each patient prior to study enrollment. The inclusion and exclusion criteria of this study are presented in Table 1.

**Table 1:** Main eligibility criteria

Inclusion criteria	Exclusion criteria
1. Female 2. Age ≥ 18 years 3. Pathologically confirmed HER2-positive invasive breast carcinoma (stage I-IV) 4. Anticipated treatment with HER2-targeted therapy for ≥ 12 months 5. Normal LV systolic function (LVEF ≥ institutional lower limit of normal) 6. Willing and able to provide written informed consent and comply with the requirements of the protocol	1. Anticipated treatment with anthracycline chemotherapy 2. Prior treatment with anthracycline chemotherapy 3. History of cardiomyopathy, heart failure, or other clinically significant cardiovascular disease 4. Uncontrolled hypertension, defined as a systolic blood pressure ≥ 160 mmHg and/or diastolic blood pressure ≥ 90 mmHg

### Clinical evaluations

**Fig. 1:**
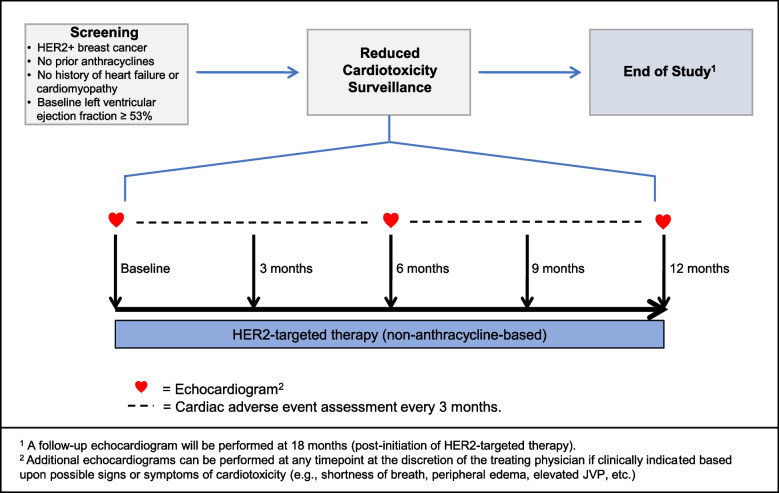
Study schema

Baseline demographics, vital signs (height, weight, systolic blood pressure, diastolic blood pressure, and heart rate), concomitant medications, cardiovascular risk factors, and cardiovascular history will be recorded at baseline upon study enrollment. Standard-of-care clinical evaluations including vital signs, interval history, and physical examination will be performed by the treating medical oncologist during HER2 targeted therapy (months 3, 6, 9, and 12) to assess for cardiac adverse events (Fig. 1).

### Echocardiography

All patients will undergo standard echocardiograms in accordance with the American Society of Echocardiography (ASE) recommendations at the following timepoints: baseline (within 3 months prior to initiating HER2-targeted therapy), and 6-, 12-, and 18-months post-initiation of HER2-targeted therapy [20]. For patients receiving ongoing HER2-targeted therapy beyond month 18, additional echocardiograms will be performed as clinically indicated per the treating oncologist. Myocardial strain assessment will be performed with standard commercially available vendor independent software using speckle tracking techniques (2D Cardiac Performance Analysis, TomTec Imaging Systems, Munich, Germany), blinded to the clinical interpretation of the 2D echocardiogram. At the end of the study, all images will be analyzed by a single reader (AY) for assessment of study outcomes.

### Endpoints

The primary study endpoint is the cardiac event rate, defined by a composite of a heart failure event (New York Heart Association class III or IV) or cardiac death. The secondary endpoints are: 1) change in LVEF and GLS at 6 and 12 months after initiation of HER2-targeted therapy compared to baseline; 2) incidence of asymptomatic LVEF decline, defined by a ≥ 10% absolute reduction from baseline to < 53%; 3) incidence of HER2-targeted treatment interruption, defined by a > 6 week delay between HER2-targeted treatment doses; and 4) feasibility, defined by the proportion of patients who complete the 6- and 12-month surveillance echocardiogram, per protocol. Patients who develop heart failure or asymptomatic LVEF decline will be referred for cardiology evaluation, and HER2-targeted therapy will be interrupted at the discretion of the treating physician.

### Study procedures and data management

Screening, baseline, and follow-up study data will be collected on standardized study worksheets. All data will be stored in a secured project database.

### Statistical considerations

The proportion of patients who develop a cardiac event at 12 months will be estimated together with an exact 95% confidence interval. Based upon our preliminary data of early-stage HER2-positive patients treated with non-anthracycline trastuzumab-based regimens, the estimated cardiac event rate is 1.2% (95% CI 0.1–4.1%) [21]. The null hypothesis of the current study is that a reduced cardiotoxicity surveillance strategy is non-inferior to routine standard-of-care surveillance by a prespecified margin in the cardiac event rate of 2.9%. This non-inferiority margin corresponds to the difference between the observed cardiac event rate from our preliminary data and the upper bound of the 95% confidence interval. With 190 patients, we will have 84% power to reject the null hypothesis using a one-sided exact test with a significance level of 0.052. We will reject the null hypothesis if no more than 3 participants develop a cardiac event. If 4 or more cardiac events are observed during any point in the trial, the study will stop.

### Study approval and current trial status

The trial was approved by the Memorial Sloan Kettering (MSK) institutional review board on March 22, 2019. An institutional data and safety monitoring committee meets every 6 months to review safety data and ensure that continuation of the study is safe. On June 29, 2021 the trial was opened to accrual at Hartford Healthcare Cancer Institute (Hartford, CT), a member of the MSK Cancer Alliance. The MSK Cancer Alliance is a collaborative partnership established in 2013 between MSK and community health systems to accelerate the integration of cancer treatment advances in the community setting and expand community access to MSK clinical trials and cancer research.

## Discussion

Cardiac imaging plays a critical role in safety monitoring for cancer patients receiving cardiotoxic treatment with anthracyclines and/or HER2-targeted therapies. However, advances in the treatment landscape of HER2-positive breast cancer have led to improvements in the cardiac safety profile of current therapies, with greater use of non-anthracycline treatment regimens associated with lower cardiotoxicity. We hypothesize that a reduction in the frequency of cardiotoxicity surveillance is safe for low-risk patients receiving non-anthracycline HER2-targeted treatment. In contrast, more frequent LVEF assessments performed every 3 months may be appropriate for patients at high risk for cardiotoxicity, such as patients with poorly controlled hypertension, pre-existing cardiovascular disease (i.e., cardiomyopathy or significant valvular heart disease), or those receiving anthracycline-based treatment. Our study evaluating the cardiac safety of reduced cardiotoxicity surveillance (every 6 months) will be an important first step towards updating current practice guidelines for cardiotoxicity surveillance.

Unnecessary clinical testing which confers no meaningful benefit to patients is a widely recognized problem that contributes to a large proportion of US healthcare spending [22]. Cardiac testing is particularly subject to overuse, prompting the American College of Cardiology to publish Appropriate Use Criteria (AUC) for many cardiovascular imaging and intervention procedures such as echocardiography [23–25]. Based upon the high volume of cardiac imaging procedures performed in patients with breast cancer, a reduction in the frequency of LVEF assessments could significantly reduce healthcare resource utilization and result in cost savings. Given that the same cardiotoxicity surveillance recommendations originally established for HER2-positive breast cancer are now being applied in other treatment settings (i.e., lung or esophageal cancer), our effort to optimize the approach for cardiotoxicity surveillance will impact a growing number of cancer patients receiving HER2-targeted treatment.

There are additional concerns that overuse of cardiac imaging during cancer treatment may subject patients to direct harms. Echocardiography, which is the most common imaging modality for LVEF measurement during cancer treatment, is limited by temporal, intra- and inter-observer variability and often subject to suboptimal image quality due to breast implants, tissue expanders, or other chest wall abnormalities. These technical limitations reduce the accuracy of echocardiographic measures of cardiac function and increase the likelihood of false positive results, especially in treatment settings with a low expected prevalence of cardiotoxicity (e.g., patients receiving non-anthracycline HER2-targeted regimens). False positive findings are of clinical significance as they can lead to: (1) additional costly and unnecessary downstream testing; (2) increased patient anxiety, and (3) delays in the timely administration of effective cancer treatment, which may adversely affect cancer outcomes [26]. Considering these potential harms, strategies to reduce unnecessary cardiac testing during cancer treatment may translate to improved patient outcomes.

### Study limitations

We considered a randomized controlled trial design to test our hypothesis that a reduced cardiotoxicity surveillance strategy is non-inferior to the current standard-of-care imaging strategy, however we opted for a single-arm study design due to the shorter duration of this study design, greater efficiency, and abundance of historical control data for comparison. The primary outcome measure of clinical heart failure or cardiac death was selected to ensure that the study findings will be clinically relevant and easily interpretable. We will also collect surrogate imaging markers throughout the study, including LVEF and GLS, in order to evaluate for subclinical measures of cardiotoxicity. Trastuzumab remains the most common HER2-targeted agent for HER2-positive breast cancer treatment, however several novel HER2-targeted therapies have been introduced in clinical practice over the past few years. Although the current study is not designed to evaluate for differences in the cardiac safety of trastuzumab versus other agents, current evidence suggests that the cardiac safety profiles of newer HER2-targeted therapies are similar [27–29].

## Conclusion

This study will provide the first prospective data of a risk-based approach to cardiotoxicity surveillance. We anticipate that findings from this study will have an immediate impact to reduce the burden of unnecessary testing and its negative downstream consequences, provide insight on the optimal frequency and duration of cardiotoxicity surveillance in the evolving landscape of HER2-targeted treatment, prevent unnecessary delay or interruption of effective cancer care, and reduce inappropriate healthcare resource utilization that contributes to the rising cost of healthcare. This study will be an important first step towards updating current practice guidelines and establishing a tailored, risk-based approach to cardiotoxicity surveillance for future patients receiving HER2-targeted therapy.

## Data Availability

Not applicable.
